# Use of bioacoustics in species identification: Piranhas from genus *Pygocentrus* (Teleostei: Serrasalmidae) as a case study

**DOI:** 10.1371/journal.pone.0241316

**Published:** 2020-10-29

**Authors:** Xavier Raick, Alessia Huby, Gregório Kurchevski, Alexandre Lima Godinho, Éric Parmentier

**Affiliations:** 1 Laboratory of Functional and Evolutionary Morphology, Freshwater and Oceanic Science Unit of Research, University of Liège, Liège, Belgium; 2 Fish Passage Center, Federal University of Minas Gerais, Belo Horizonte, Minas Gerais, Brazil; University of Pavia, ITALY

## Abstract

The genus *Pygocentrus* contains three valid piranha species (*P*. *cariba*, *P*. *nattereri* and *P*. *piraya*) that are allopatric in tropical and subtropical freshwater environments of South America. This study uses acoustic features to differentiate the three species. Sounds were recorded in *P*. *cariba*, two populations of *P*. *nattereri* (red- and yellow-bellied) and *P*. *piraya*; providing sound description for the first time in *P*. *cariba* and *P*. *piraya*. Calls of *P*. *cariba* were distinct from all the other studied populations. Red- and yellow-bellied *P*. *nattereri* calls were different from each other but yellow-bellied *P*. *nattereri* calls were similar to those of *P*. *piraya*. These observations can be explained by considering that the studied specimens of yellow-bellied *P*. *nattereri* have been wrongly identified and are actually a sub-population of *P*. *piraya*. Morphological examinations and recent fish field recordings in the Araguari River strongly support our hypothesis. This study shows for the first time that sounds can be used to discover identification errors in the teleost taxa.

## Introduction

Sounds for communication purposes are emitted by a high number of different zoological groups. Many species have evolved calls that are assumed to be species-specific [1–3] but that can sometimes show intraspecific variability since age, sex, and size can affect different call parameters such as frequency and amplitude [4–11]. The specific features of calls easily explain why sounds were successfully used to differentiate species of orthopterans [12], teleosts [13], birds [14,15], bats [16,17], and cetaceans [18–20]. Bioacoustics is also used as part of the diagnosis in bird species [14] where call features complement genetic and morphological data [21–27] simply because the use of acoustic signals is far easier to distinguish the species in the field. To the best of our knowledge, the consolidation of a fish species diagnosis with acoustic data has only been done in *Encheliophis chardewalli* (Carapidae) in which the comparison with other sympatric species of the same tribe supports a distinct repertoire [28].

“Cryptic” or “twin-species” are species that cannot be separated using characters of external morphology [29,30]. “Cryptic” species are mainly found in taxa that communicate reproductive signals via nonvisual cues [30]. Acoustic mating signals were thus effective in discriminating cryptic species in different invertebrates and vertebrates [30]. This kind of investigation is only suggested in teleosts [31]. For example, the cusk-eels *Ophidion barbatum* and *O*. *rochei* possess almost the same external phenotype [32] but show remarkable differences between their sound-producing apparatuses, supporting the production of different kinds of calls [32–34]. In electric fish, cryptic species were discriminated using features of their electric organ discharges [35].

**Fig 1:**
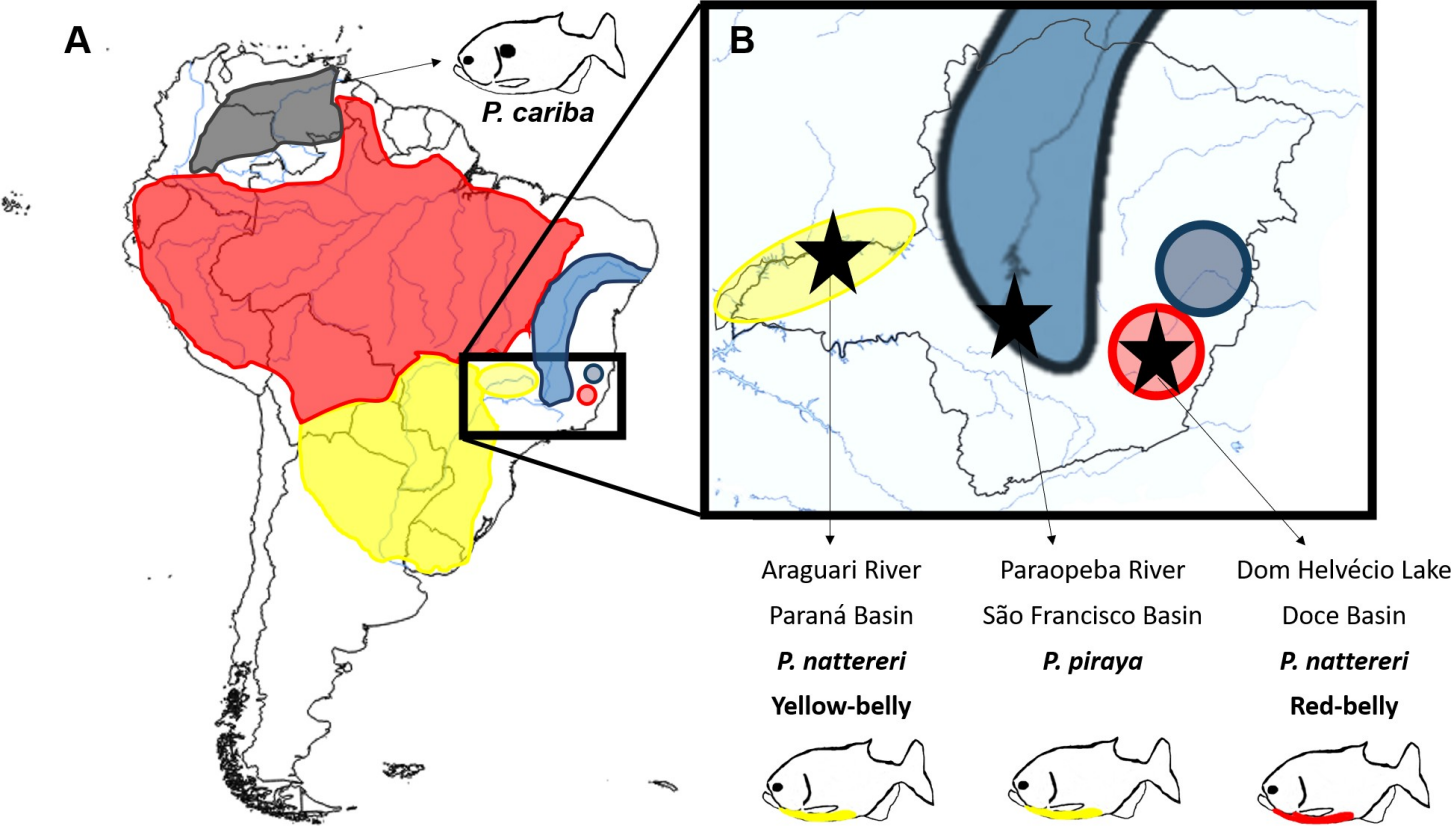
A. Distribution of the different species of genus *Pygocentrus* modified from [48] and adapted with data from non-native populations [39–41,49]. The range of *P*. *cariba* is represented in black, that of *P*. *nattereri* is approximately divided into red and yellow according to the colouration of the belly and the river basins, and that of *P*. *piraya* is in blue. B. Locations of sampling sites (black stars) in Minas Gerais state, Brazil.

Serrasalmidae includes pacus, silver dollars and piranhas [36,37]. Among piranha species, the *Pygocentrus* genus has three valid species: *P*. *cariba*, *P*. *nattereri*, and *P*. *piraya*. In addition, *P*. *palometa*, found in the Orinoco River basin, was described by Valenciennes in 1850 but is currently considered *nomen dubium* [38]. The three valid species are allopatric (Fig 1A). *Pygocentrus cariba* occurs in the Orinoco River basin (Northern South America) while *P*. *piraya* is restricted to the São Francisco River basin (Eastern South America). *Pygocentrus nattereri* has a wider distribution since it can be found in nine countries in South America between the areas inhabited by both previous species. It can be found in the watershed of the Essequibo, Amazon, and Paraná-Paraguay rivers, as well as in the north-eastern Brazilian coastal river basins [38]. Moreover, it has been introduced in many places, for example, the lakes of the Doce River [39–41]. Contrary to the two other species, *Pygocentrus cariba* has a black humeral spot. *Pygocentrus piraya* and southern populations of *P*. *nattereri* possess a yellow belly whereas *P*. *nattereri* from other locations all possess a red belly [42,43].

In piranhas, *Pygocentrus* and *Serrasalmus* possess a well-developed sound-producing apparatus that is used in acoustic communication [10,44] and therefore plays a role in their ecology [45]. These sounds are warning calls [10] and are identical to those recorded during frontal displays [46]. Behavioural studies have shown that these calls are produced 80% of the time during frontal display between two fish and 20% of the time, these sounds could not be associated with a given behaviour [46]. They are species-specific, harmonic and generally made of continuous peaks with a low fundamental frequency below 150 Hz [10,46,47]. This study aimed to use acoustic features to differentiate the three current valid *Pygocentrus* species, providing sound description for the first time in *P*. *cariba* and *P*. *piraya*. In addition, this study also investigates the difference between yellow- and red-bellied populations of *P*. *nattereri*.

## Materials and methods

### Biological materials

Sounds were recorded from specimens maintained in aquaria and from specimens captured in the wild (Table 1). Three specimens of *Pygocentrus cariba* (standard length, SL: 90 ± 2 mm; mean ± standard deviation) and five specimens of *P*. *piraya* (SL: 98 ± 8 mm) were purchased in an exotic import aquarium store (Table 1). They were housed at the University of Liège (Belgium) in tanks (volume ≥ 100 L) with external filters, internal heaters (26 ± 1°C), bubblers and light (12 h light/dark cycle). They were fed three times a week with *Mytilus edulis*. Four additional specimens of *P*. *piraya* (SL: 409 ± 19 mm) from the aquarium Aquário da Bacia do Rio São Francisco of Belo Horizonte were used for sound recordings (Table 1). We did not use specimens of *P*. *nattereri* from aquarium stores because for this species there is a lot of mixing of specimens from different river basins and a lot of artificial selection.

**Table 1:** Specimens recorded in this study.

Species	Variety	Localisation	N (fish)	N (sounds)
*Pygocentrus cariba*		University of Liège (origin: São Francisco River Basin)	3	26
*Pygocentrus piraya*		University of Liège (origin: Orinoco River Basin)	5	50
		Aquário da Bacia do Rio São Francisco (Belo Horizonte)	4	0
		Paraopeba River	3	30
*Pygocentrus nattereri*	Red-bellied	Parque Estadual do Rio Doce	150	1500
	Yellow-bellied	Araguari River	11	107

Additional specimens and species were fished in Brazil in July 2018 (Table 1). Three specimens of *P*. *piraya* (SL: 185 ± 86 mm) were captured with gillnets and by hook-and-line in the Paraopeba River (18°52′35″ S, 44°46′49″ W; São Francisco River Basin). Two populations of *P*. *nattereri* were studied: red-bellied and yellow-bellied. Red-bellied *P*. *nattereri* were from Dom Helvécio Lake (Parque Estadual do Rio Doce; 19°46′29″ S, 42°35′57″ W; Doce River Basin) where 150 specimens (SL: 178 ± 24 mm) were captured with gillnets. Specimens of yellow-bellied *P*. *nattereri* were from Araguari River (18°39′37″ S, 48°26′20″ W; Upper Paraná River Basin) where eleven specimens (SL: 328 ± 18 mm) were captured with gillnets and by hook-and-line. There was no effect of the fishing method on the sounds recorded. There is no geographical natural connection between red-bellied and yellow-bellied *P*. *nattereri*. Pictures of a specimen from each geographical group can be found in S1 Fig. Some specimens are available at the Aquarium-Museum of the University of Liège (vouchers R.E. 15271, 15272, 15273, 15274, 15275 and 15276).

### Recordings & sound-analysis

The sounds were recorded in a glass-tank (108 L), with a hydrophone (HTI-96-Min, High Tech Inc., USA; sensitivity: –164.4 dB re 1V μPa^-1^) placed in the middle of the aquarium and connected to a TASCAM DR5 recorder (TEAC Corporation, USA). The fish were gently hand-held between the thumb and the index of the left hand without any pressure at approximatively five centimetres from the hydrophone in order to homogenize as much as possible the sounds recorded. During recordings in the University of Liège, the water temperature was 26 ± 1°C and the filters, heater, bubblers and the light of the aquarium were turned off to reduce non-biological noises. For the specimens recorded in Brazil, the water temperature in the aquarium was 26 ± 2°C. The resonance frequency was 3252 Hz (for details on recordings in tanks, see [50]).

In total, 1713 sounds were recorded and analysed. For each vocal specimen, ten sounds were analysed, except for *P*. *cariba* and yellow-bellied *P*. *nattereri* from the Araguari River for which 26 and 107 sounds have been analysed, respectively. The sounds were digitised on mono-channel at 44.1 kHz with a 16 bit-resolution and then they were sub-sampled at 4000 Hz and a high-pass filter at 50 Hz was applied. The analysis was manually carried out with Avisoft SAS-Lab Pro 5.2 (Avisoft Bioacoustics, Germany).

**Fig 2:**
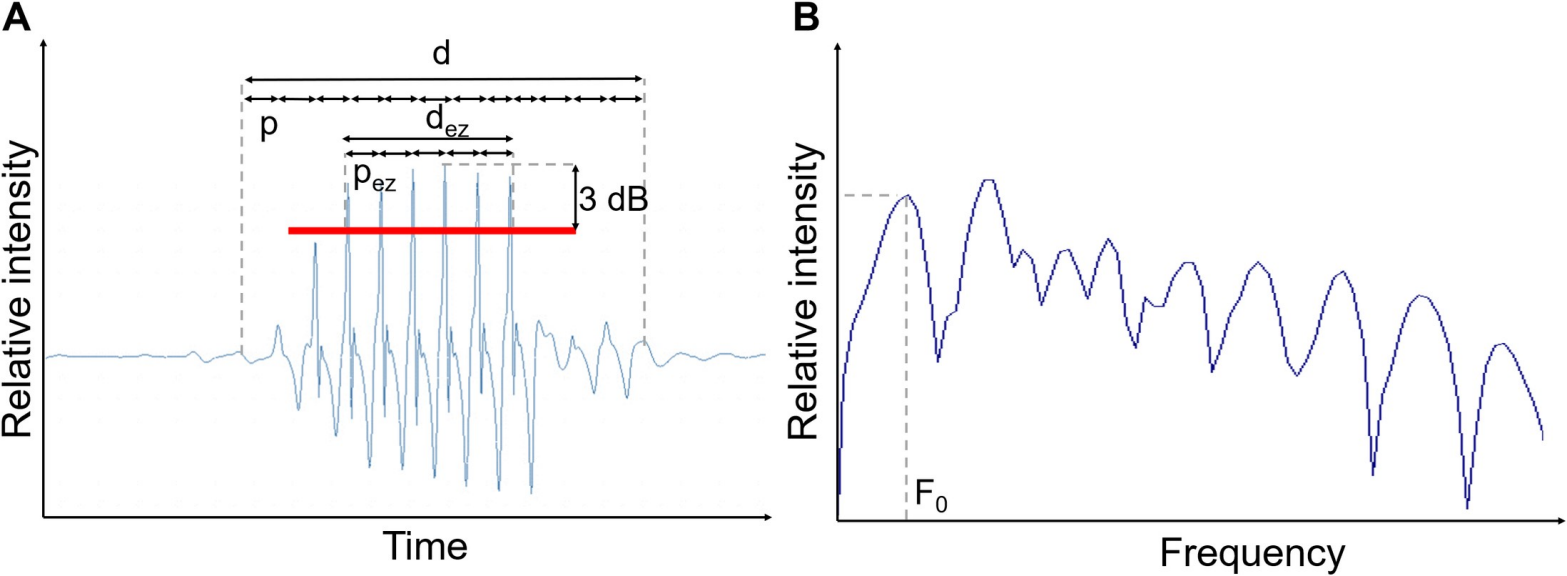
A. Oscillogram and B. power spectrum of a sound with the features measured. d = duration, d_ez_ = duration of the energetic zone, p = period, p_ez_ = period in the energetic zone, F_0_ = fundamental frequency.

The temporal features were measured on oscillograms (Fig 2A) while the fundamental frequency (**F**_**0**_, in Hz) was obtained from the power-spectra of the sounds (Fig 2B). The temporal features were the sound duration (**d**, in ms); the number of peaks in a sound (**N**), and the period between consecutive peaks (**p**, in ms). The period of the first peaks has been shown to be longer than that of the rest of the sound in some piranha species [10]. In consequence, the same three features were measured in the most energetic zone (abbreviated “**ez**”), i.e., the continuous area with an intensity of maximum 3 dB less than the maximum intensity peak (as the dB scale is a logarithmic scale, it corresponds to a decrease of a factor 1.4, see Fig 2A) and named **d**_**ez**_, **N**_**ez**_, and **p**_**ez**_.

### Statistics

The description of the sounds was carried on the totality of the sounds. For each species, the different features were compared using Wilcoxon signed-rank tests and correlations between features were calculated using Bland-Altman repeated measures correlations.

The comparison between the different species required knowledge of the effect of the fish size because this could affect acoustic features [10]. For this reason, the effect of size was removed in order to conduct suitable comparisons. All the statistics relative to the effect of body size and the species comparison were realised on means per individuals, i.e., the ten sounds were averaged to obtain a single value per specimen. Correlation matrices were calculated using Spearman correlation coefficients (r_S_) and their associated P-value matrices corrected by the Holm-Bonferroni method. When a correlation between an acoustic feature and the SL was found, a linear regression was performed. Logarithmic and polynomial regressions were also tested.

Wilcoxon-Mann-Whitney tests (also named Mann-Whitney U tests) and Kruskal-Wallis tests, with the Dunn’s test as *post-hoc* with a Benjamini-Hochberg correction on the *P*, were used to compare acoustic features between species. The values of the statistics W and Z are presented with *P* and adjusted *P* when necessary. To reduce the multivariate aspect of the data, principal component analyses (PCAs) were conducted on the correlation matrices with all acoustic features except those whose R^2^ ≥ |0.60|, for which the features divided by the SL were used instead in order to remove the effect of size. In parallel, we carried out the same analyses on the residuals of the linear regressions. As the statistical significances and the biological conclusions with the two methods were identical, we present only the first method in this manuscript. We did not perform phylogenetic corrections because the number of species was small and it would have not been possible to take into account the difference between red- and yellow-belly *P*. *nattereri*. The principal components (PCs) are presented with their associated percentage of variance and their cumulative percentage of variance. PCs for each group were compared using Wilcoxon-Mann-Whitney and Kruskal-Wallis tests with the Dunn’s test as *post-hoc*. All the statistics were carried-out using R 3.3.0. (GNU General Public License) and the significance level was α = 0.05.

### Authorisations

The experiment was approved by the ethical commission of the University of Liège (case 1532) and the capture of the Brazilian specimens was achieved under the license 10306–1 from the Brazilian Ministry of the Environment. In the Paraopeba River, the fish were captured near the Retiro Baixo Hydropower Dam with an authorisation n° PT-04/07/2018 issued by Retiro Baixo Energética. In the Parque Estadual do Rio Doce, the fish were sampled under the license 049/2018 from the Instituto Estadual de Florestas of the State of Minas Gerais. In the Araguari River, the fish were captured near the Capim Branco II Hydropower Dam with an authorisation from Consórcio Capim Branco. Six specimens have been euthanasied with an overdose of eugenol (CAS: 97-53-0) and imported to Belgium with the authorisation of the Federal Agency for the Safety of the Food Chain (COBT/IEC/CMY/1546595), the Federal University of Minas Gerais (MTA/TTM n° 02/2019) and the Conselho de Gestão do Patrimônio Genético of the Ministério do Meio Ambiente (n°R2B8FF7 et n°A57A7E9).

## Results

### Description of the sounds

Sounds of *P*. *cariba*, two populations of *P*. *nattereri* (red- and yellow-bellied) and *P*. *piraya* were recorded in this study. Sonic muscles were similar in the three species. All specimens produced sounds except four specimens of *P*. *piraya* coming from the Aquário da Bacia do Rio São Francisco of Belo Horizonte, for which body size was between 390 and 430 mm.

#### Description of *Pygocentrus cariba* sounds

**Fig 3:**
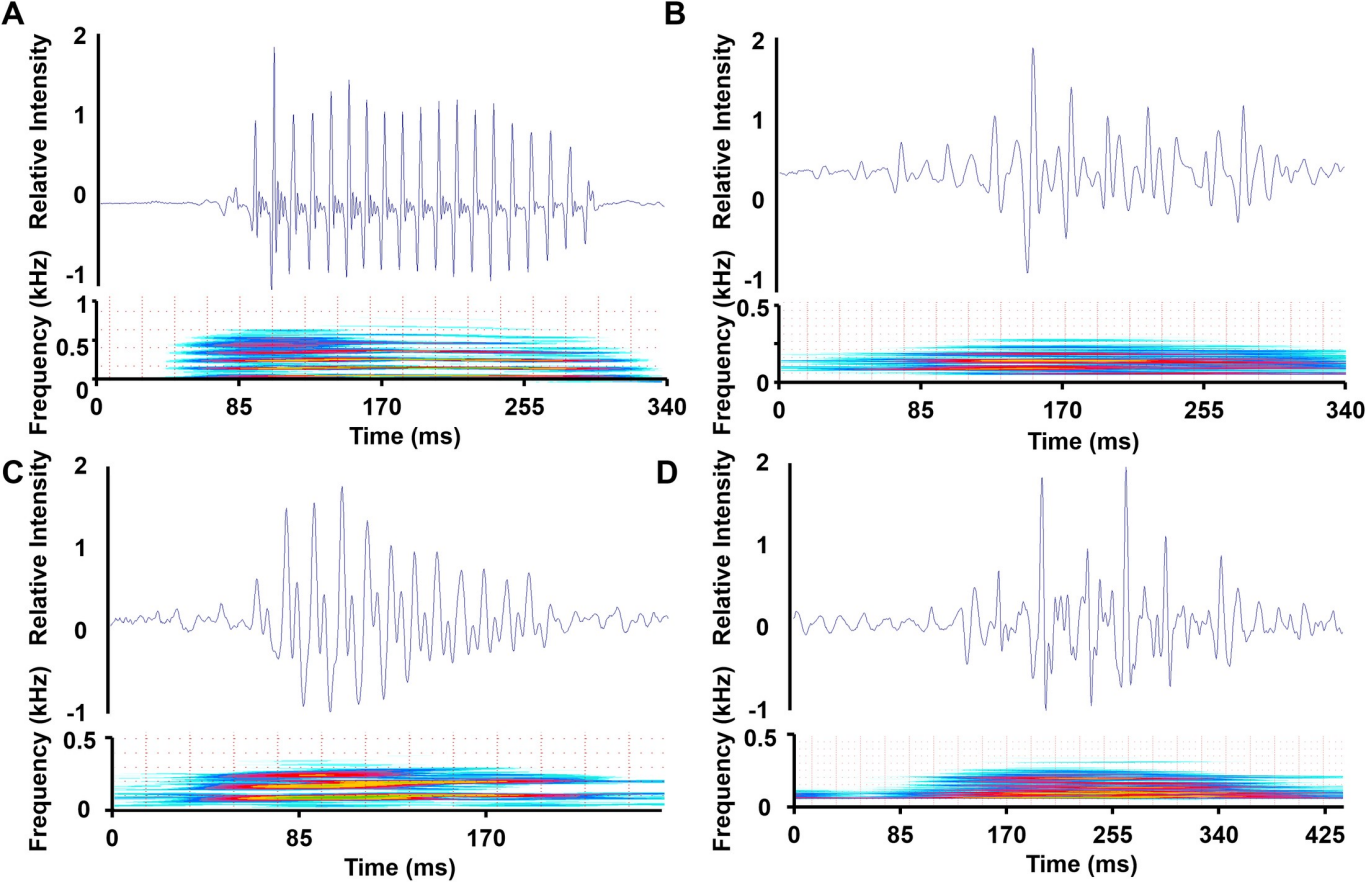
Waveform (top) and spectrogram (bottom) of a sound produced by A. *Pygocentrus cariba*, B. *Pygocentrus piraya*. C. red-bellied *Pygocentrus nattereri* and D. yellow-bellied *Pygocentrus nattereri*. FFT Length: 512, window: Hamming, frame: 100%.

*Pygocentrus cariba* SLs were between 87 and 92 mm. Their sounds (n_sounds_ = 26) were low-frequency harmonic drumming sounds and consisted of 16 ± 7 (mean ± standard deviation) continuous peaks with a peak period of 11.5 ± 0.5 ms (Fig 3A). Sounds lasted from 54 to 388 ms (183 ± 74 ms) with a fundamental frequency of 89 ± 6 Hz. The most energetic zone contained between 2 and 18 peaks (7 ± 5 peaks) that corresponded to 15 to 80% (45 ± 16%) of the total number of peaks. The peak period in the most energetic zone was equivalent to the mean peak period of the sound (Wilcoxon signed rank test: V = 208, *P* = .42). The sound duration was positively correlated with the number of peaks (r = 0.99, *P* < .0001) but not with the period (r = –0.22, *P* = .30). In other words, the number of peaks determines the sound duration. **N**_**EZ**_ and **d** were correlated with **d**_**EZ**_ (r = 0.94 and 0.78, both *P* < .0001).

#### Description of *Pygocentrus piraya* sounds

*Pygocentrus piraya* SLs were between 90 and 281 mm. Their sounds (n_sounds_ = 80) were made of 9 ± 3 continuous peaks with a peak period of 15 ± 5 ms (Fig 3B). Sounds lasted from 71 to 266 ms (131 ± 50 ms) with a fundamental frequency of 79 ± 13 Hz. The most energetic zone of *P*. *piraya* sounds was much smaller than in *P*. *cariba* sounds (34 ± 30 ms vs. 94 ± 63 ms) with 4 ± 2 peaks and covered 38 ± 17% of the total number of peaks. The period in the energetic zone was 14 ± 5 ms. The number of peaks was independent of the duration of the energetic zone (r = 0.16, *P* = .18) and weakly correlated to the number of peaks in the energetic zone (r = 0.24, *P* = .04).

#### Description of *Pygocentrus nattereri* sounds

The specimens of yellow-bellied *P*. *nattereri* were much bigger than the specimens of red-bellied *P*. *nattereri* (Wilcoxon-Mann-Whitney: W = 0, *P* < .0001). Red-bellied *P*. *nattereri* sounds (N_sounds_ = 1500) were made of 11 ± 3 continuous peaks with a peak period of 14 ± 2 ms. Sounds lasted from 24 to 417 ms (147 ± 43 ms) with a fundamental frequency of 79 ± 12 Hz. The most energetic zone contained between 1 and 23 peaks (4 ± 2 peaks) that corresponded to 42 ± 16 of the total number of peaks (Fig 3C). Yellow-bellied *P*. *nattereri* sounds (N_sounds_ = 107) were made of 6 ± 3 continuous peaks with a peak period of 31 ± 8 ms. Sounds lasted from 40 to 466 ms (194 ± 90 ms) with a fundamental frequency of 70 ± 9 Hz. The most energetic zone contained between 1 and 7 peaks (2 ± 1 peaks) that corresponded to 36 ± 22% of the total number of peaks (Fig 3D).

### Effect of the fish size

**Fig 4:**
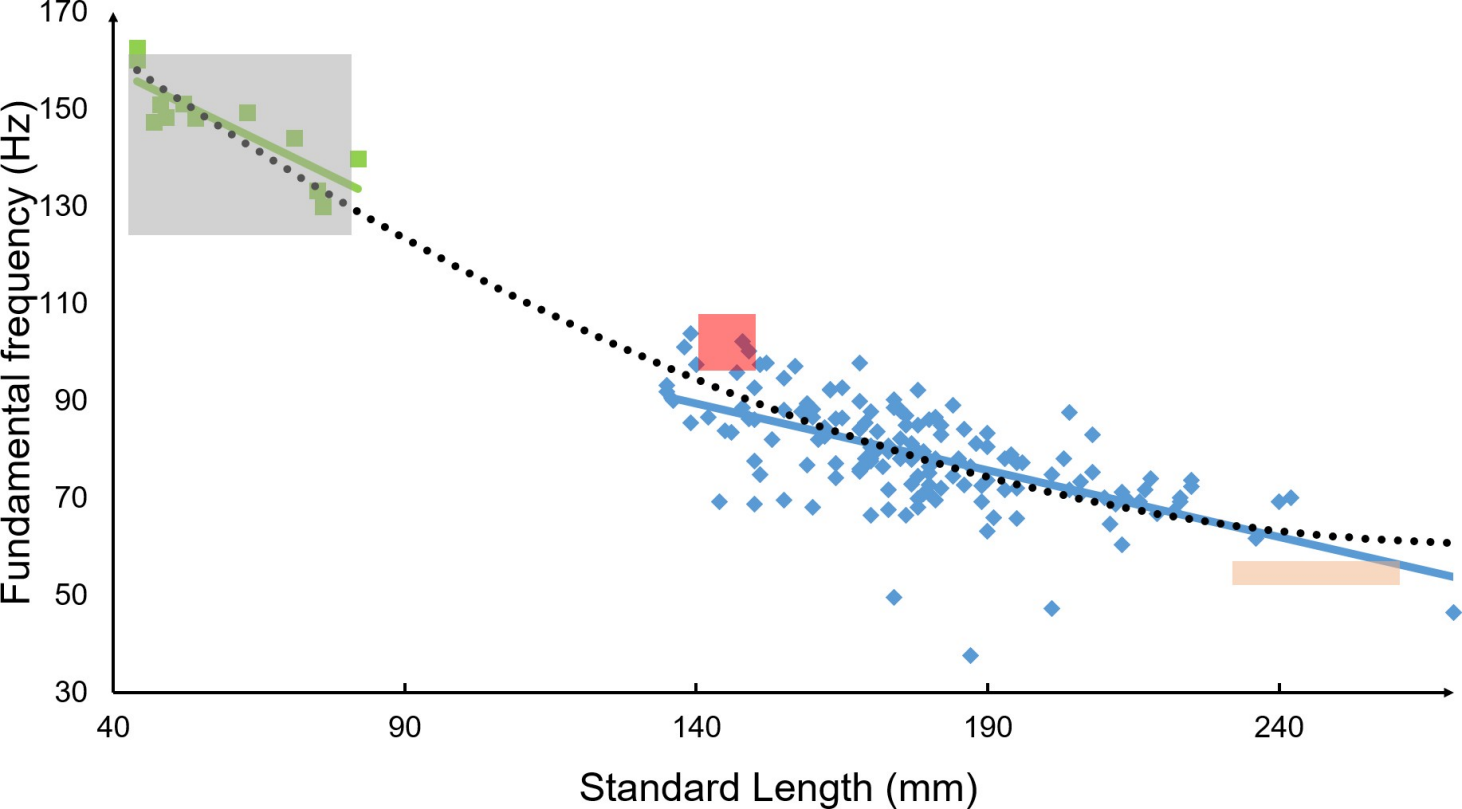
Fundamental frequency as a function of the standard length for red-bellied *Pygocentrus nattereri*. The three boxes show the data from [55]. Each box corresponds to the mean ± SD of a given size class. The green “■” are from [10] and the blue “♦” from this study. Linear regressions are presented for separated datasets and a polynomial model of all the data.

For all species, all the frequency features and the peak periods were correlated to the SL (all *P* < .001). The **p** and **p**_**ez**_ were highly positively correlated to the SL (r_S_ = 0.64 & 0.73) while the fundamental frequency was highly negatively correlated to the SL (r_S_ = –0.62). When **p** and **p**_**ez**_ were predicted, it was found that the SL was a significant predictor (both *P* < .001, both R^2^ = 0.68). To remove the effect of the size, the features divided by the SL (written “**X SL**^**-1**^” for the feature “**X**”) were used instead of the features themselves. In fish, some sound features, such as pulse duration or fundamental frequency, are known to be affected by their body size [8,9,51–54]. We combined our results with data also recorded in glass tanks from [55] and [10] to study the frequency as a function of size with 182 different specimens of red-bellied *P*. *nattereri* ranging from 44 to 270 mm (Fig 4). Besides linear regression, other models such as logarithmic models (Table 2) could explain the relation between the size and the fundamental frequency, with larger specimens having a smaller fundamental frequency.

**Table 2:** Adjustment models with their respective R^2^ for the fundamental frequency as a function of the standard length in red-bellied *Pygocentrus nattereri*.

Type of model	Equation	R^2^
Linear	y = –0.50 x + 168.96	0.84
Logarithmic	y = –59.79 ln (x) + 388.77	0.88
Polynomial	y = 0.0018 x^2^–0.99 x + 198	0.88

### Species discrimination

**Fig 5:**
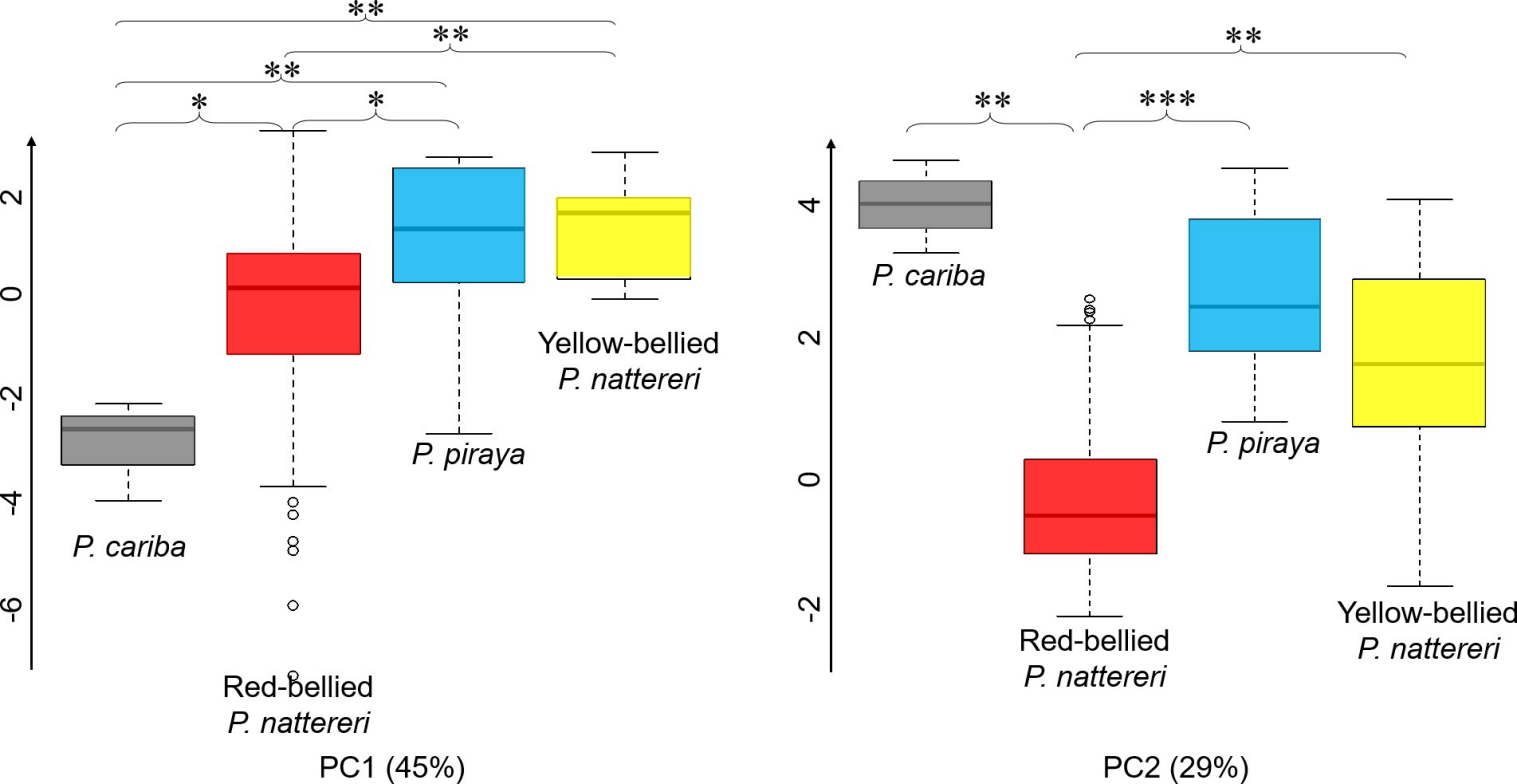
Comparison of the scores from principal component analysis (PCA) in the genus *Pygocentrus*. Boxes represent the median ± interquartile range (IQR) and lines represent Q1–1.5 IQR and Q3 + 1.5 IQR. * = *P* < .05; ** = *P* < .01; *** = *P* < .001; Kruskal-Wallis + Dunn.

**Fig 6:**
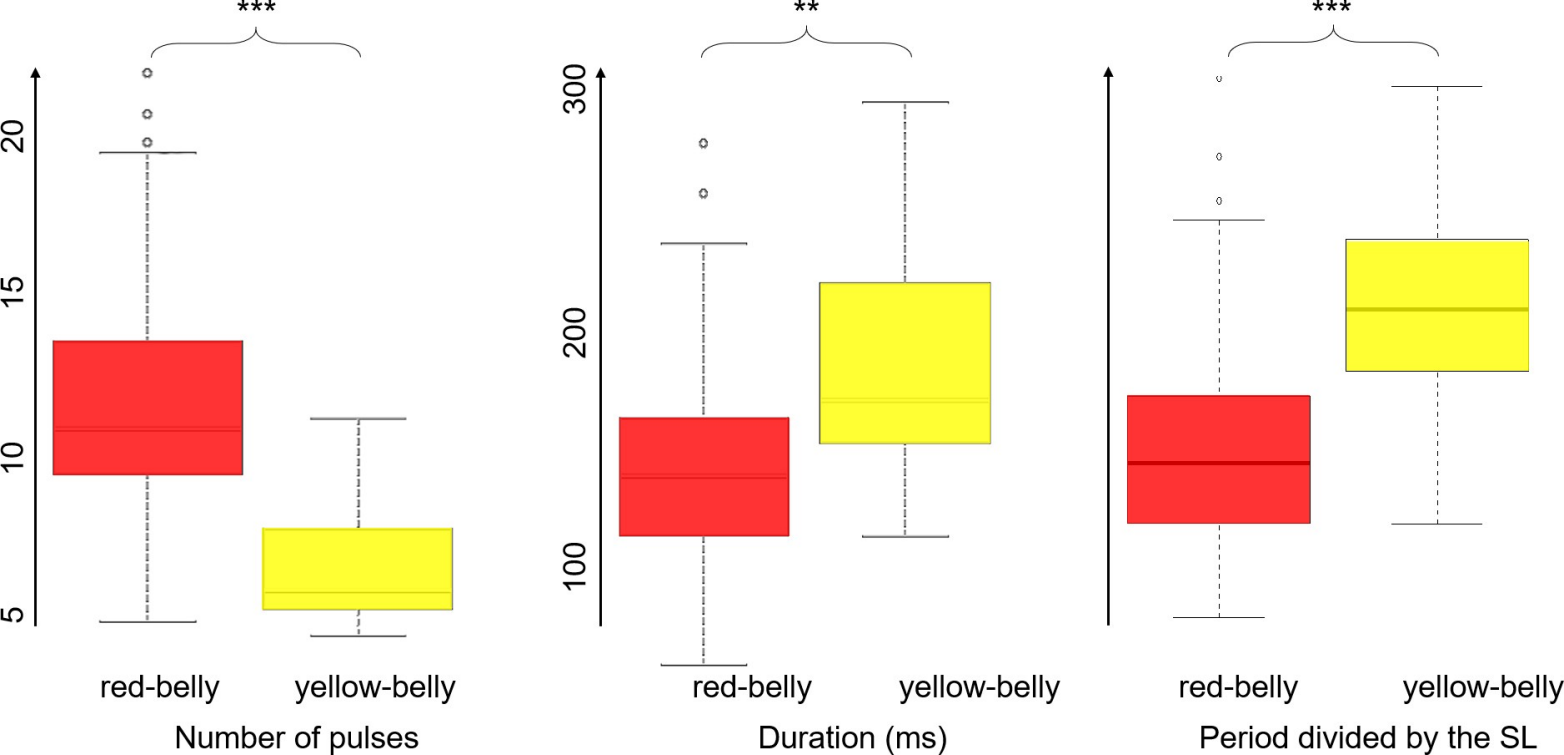
Comparison of acoustic features of red and yellow-bellied *Pygocentrus nattereri*. Boxes represent the median ± interquartile range (IQR) and lines represent Q1–1.5 IQR and Q3 + 1.5 IQR. ** = *P* < .01; *** *P* < .001; Wilcoxon-Mann-Whitney.

The **d**_**ez**_, **N**, and **N**_**ez**_ of the sounds of *Pygocentrus cariba* were different from those of all the other species (Kruskal-Wallis: χ^2^ = 10, 29 & 30; df = 3; *P* = .02, < .001 & < .001; Dunn: Z = 2.64, 3.06 & 2.80; *P* = .02, .01 & .02 for d_EZ_, Z = 2.43, 3.00 & 4.30; *P* = .02, .005 & < .001 for N, Z = 2.22, 2.71 & 4.25; *P* = .04, .01 & < .001 for N_EZ_). The two features **p SL**^**-1**^ and **p**_**ez**_ **SL**^**-1**^ were the only ones that separated the red-bellied *P*. *nattereri* from all the others (Kruskal-Wallis: both χ^2^ = 38, df = 3, both *P* < .001; Dunn: Z = 3.08, –4.47 & –3.36; *P* = .004, < .001 & .002 for p SL^-1^ and Z = 3.12, –4.66 & –3.18; *P* = .004, < .001 & .004 for p_ez_ SL^-1^)and were equivalent for all the other comparisons (Dunn: Z = 0.26, 1.15 & 1.23; *P* = .80, .30 & .33 for p SL^-1^ and Z = .18, 1.27 & 1.51; *P* = .85, .25 & .20). The PCA performed on all the features (or on the features divided by the SL when applicable) showed that PC1 (45%) values were statistically different between all the groups (Kruskal-Wallis: χ^2^ = 19, df = 3, *P* < .001; Dunn: Z = –2.30, –3.12, –2.12, –3.49 & –2.98; *P* = .03, .005, .04, .003 & .006)) except between *P*. *piraya* and yellow-bellied *P*. *nattereri* (Dunn: Z = –0.35, *P* = .73). PC1 was highly negatively correlated with **d**, **d**_**ez**_, **N**, **N**_**ez**_ (r_S_ = –0.66, –0.75, –0.90 & –0.86; all *P* < .001). PC2 (29%, 74% cumulative) separated red-bellied *P*. *nattereri* from all the others (Table 3 and Fig 5). The comparison between the studied specimens of red-bellied and yellow-bellied *P*. *nattereri* showed that their sounds differ on all the features (Wilcoxon-Mann-Whitney: W = 401, 1328, 1246, 1486, 1534, 0, 6; *P* = .004, < .001, .005, < .001, < .001, < .001 & < .001; Fig 6) except **d**_**ez**_ (Wilcoxon-Mann-Whitney: W = 962, *P* = .36). Similar results were obtained after dividing by the SL. No difference was found between the acoustic features of yellow-bellied *P*. *nattereri* and *P*. *piraya*.

**Table 3:** Scores of the Dunn *post-hoc* test on the principal components. Significant *P*-values in red.

Species/variety comparison	Z		Non adjusted *P*		Adjusted *P*	
	PC1	PC2	PC1	PC2	PC1	PC2
*P*. *cariba* vs. red-bellied *P*. *nattereri*	–2.30	3.13	.02	.0017	.03	.003
*P*. *cariba* vs. *P*. *piraya*	–3.12	0.34	.002	.73	.005	.73
Red-bellied *P*. *nattereri* vs. *P*. *piraya*	–2.12	–4.40	.034	< .001	.04	< .001
*P*. *cariba* vs. yellow-bellied *P*. *nattereri*	–3.49	1.10	< .001	.27	.003	.40
Red- vs. yellow-bellied *P*. *nattereri*	–2.98	–3.55	< .001	< .001	.006	.001
*P*. *piraya* vs yellow-bellied *P*. *nattereri*	–0.35	1.05	.73	.29	.73	.35

## Discussion

This study described for the first time sounds of hand-held *P*. *cariba* and *P*. *piraya*. These results are important for future passive acoustics monitoring (PAM) of those species. However, caution need to be taken when compared fish sounds recorded in tanks (see [50] for details). Another important consideration is the fish size effect. Non-linear regression models has also been suggested in Balistidae [8,56] but with fewer specimens. In red-bellied *P*. *nattereri*, old adult specimens would be mute [55] because sonic muscles have been invaded by fat cells and there are several modifications in the ultrastructure of the myofibrils and sarcoplasmic reticulum [55]. In the present study, the four biggest specimens of *P*. *piraya* did not produce any sounds, supporting the hypothesis that older piranha specimens lose their sound-producing ability. Deep analysis of sonic muscles from these specimens did not take place because these fish were from a public aquarium and were returned after this experiment.

The challenge of this study was to discriminate three piranha species using their sound features. The easiest to discriminate was *P*. *cariba* which differs from all the others in **d**_**ez**_, **N**, and **N**_**ez**_. The greater differences in acoustic features between *P*. *cariba* and congeners could be related to their higher genetic distance [57,58]. *Pygocentrus cariba* and the potential ancestor of both the other sister species (*P*. *nattereri* and *P*. *piraya*) would have separated around 8 Ma, whereas *Pygocentrus nattereri* separated from *P*. *piraya* only more recently [48,57], at 2.63 ± 0.2 Ma [57–59]. Interestingly, the studied specimens of yellow-bellied *P*. *nattereri* and *P*. *piraya* cannot be distinguished using acoustic features, whereas the studied specimens of yellow-bellied and red-bellied *P*. *nattereri* can. This observation does not correspond to the theory that sounds are species-specific [60–63]. This paradox could be solved with two complementary hypotheses: yellow-bellied and red-bellied *P*. *nattereri* are two different species and yellow-bellied *P*. *nattereri* have been wrongly identified, being in fact *P*. *piraya*.

The current yellow-bellied *P*. *nattereri* was first described as *Serrasalmo ternetzi* by Steindachner in 1908 [64] before being considered the holotype of the *Gastropristis* genus by Eigenmann 1915 [65,66]. The main distinctive feature was the morphology of the anal fin. Unfortunately, the holotype was mutilated and is lost to science [42]. This yellow-bellied population of piranha was then described as *Serrasalmus ternetzi* [67] based on three specimens that differ from *P*. *nattereri* at the level of the anterior profile, the length of the dorsal-fin base and the distance of dorsal fin from adipose fin [42,67]. Subsequent studies on a higher number of individuals confirmed the status of *S*. *ternetzi*. The diagnosis used the yellow coloration (on about 100 specimens), the convexity of the head and other meristic data in thirteen specimens [42,68]. However, using the body shape and head convexity of 60 specimens, Fink (1993) considered *S*. *ternetzi* as a junior synonym of *P*. *nattereri* [42]. More recently, a study observed differences in meristic data from north and south populations of *P*. *nattereri*: yellow-bellied *P*. *nattereri* possessed a deeper body relative to body length than red-bellied *P*. *nattereri* [43]. They considered this species as a nonlinear cline [43] and like in our study, their specimens of yellow-bellied *P*. *nattereri* were again bigger than those of red-bellied *P*. *nattereri*. Molecular studies show that specimens from different geographical areas (the red-bellied from Amazonas and yellow-bellied from Paraná-Paraguay) have exclusive haplotypes, supporting that a split happened at around 1.8 Ma [48,57,69]. Morphological, meristic, colour pattern, and molecular data all support deep differences between yellow-bellied and red-bellied *P*. *nattereri*. On the studied specimens, acoustic features clearly reinforce this assertion.

According to Fink (1993), “*Pygocentrus piraya* is diagnosed by the presence of adipose fin rays and is restricted to the Rio São Francisco of Brazil” whereas “*P*. *nattereri* is undiagnosed and highly variable in pigmentation” [42]. Fink (1993) reported that *P*. *nattereri* can be distinguished from *P*. *cariba* by the lack of a black humeral spot and from *P*. *piraya* by a lack of adipose fin rays [42]. We did not observe black humeral spots but we observed adipose fin rays in the so-called yellow-bellied *P*. *nattereri* (S1 Fig) sampled in the Araguari River (Paraná River basin). However, *Pygocentrus* is not native to this location [70,71] and was recorded for the first time in 2009 (S2 Fig; Godinho, personal data) [72–74] and identified as yellow-bellied *P*. *nattereri* by Langeani & Lacerda-Rêgo (2014) [71]. According to the characters they use in their study, it is however not possible to precisely identify the *P*. *nattereri* species but only the *Pygocentrus* genus. According to our results, having a population of yellow-bellied *P*. *nattereri* in Araguari River is deeply questionable and could result from misidentification. Based on historical morphological description and on the main argument that they both have the same acoustic features, the so-called yellow-bellied *P*. *nattereri* of the Araguari River should be *P*. *piraya*. Yearly records show a progressive migration from East to West (S2 Fig), suggesting fish were introduced to the east part of Araguari River [49] (S2 Fig; Godinho, personal data). This greatly supports our sonic-based assertion: *P*. *piraya* are now found in the Paraná River basin.

Our study supports that acoustic features can be important to reveal misidentified fish species. Up to now, this tool has been only used in other zoological groups. The distinction between species appears to be possible because acoustic signalling may be more flexible than morphology [17]. In birds, sounds were used for the distinction of three species of Phylloscopidae 30 years before their official description as different species [75]. Songs were also used to clarify systematic relationships in Passeriformes [76] and to contribute to the description of new species in Rhinocryptidae [77] and Tyrannidae [78]. Insects (Orthoptera, Hemiptera, and Neuroptera) [79–81] and anurans (Leptodactylidae, Ceratobatrachidae, Ranidae) [82–88] also possess many cryptic species that can be mainly differentiated acoustically. This role of bioacoustics in taxonomy supports the application of PAM in conservation programmes. However, more studies on frequency discrimination by fish are still necessary to know if acoustic differences are distinguishable by the fish.

## Conclusions

Calls of *P*. *cariba* (Orinoco River basin) were distinct from all the other, red- and yellow-bellied *P*. *nattereri* (Parque Estadual do Rio Doce and Araguari River respectively) calls were different, and yellow-bellied *P*. *nattereri* calls were similar to those of *P*. *piraya* (São Francisco River basin). As the yellow-bellied *P*. *nattereri* found in the Araguari River basin possess adipose fin rays and the same acoustic features as *Pygocentrus piraya*, we have to consider that the population from Araguari River is most probably *Pygocentrus piraya*. This means that the geographical area of *Pygocentrus piraya* is larger than expected; and more likely, *P*. *piraya* has been introduced to the Paraná River basin. The use of acoustic features was made possible due to the removal of the effect of the fish size.

## Supporting information

S1 Fig Pictures of a studied specimen of Pygocentrus cariba (A), P. piraya (B), red-bellied P. nattereri (C) and yellow-bellied P. nattereri (D) and a magnification of its adipose fin showing adipose fin rays (E). Millimetre paper (10 x 10 mm) as scale.
(TIF)

S2 Fig A. Minas Gerais and B. São Francisco River basin (in blue) and Paraná River basin (in yellow) with the sites and dates of first observations of Pygocentrus in the upper Paraná River basin.
(TIF)

S1 Table Acoustic features of the sounds of the three valid species of *Pygocentrus*. SD = standard deviation, SE = standard error, IQR = interquartile range, CV = coefficient of variation, Min = minimal value, Max = maximal value. Acoustic features symbols as defined in Fig 2. Body size (standard length) is given under each species name.
(DOCX)
