# Supplementary figures and images for: Use of bioacoustics in species identification: Piranhas from genus Pygocentrus (Teleostei: Serrasalmidae) as a case study
Source: PLoS One. 2020 Oct 29;15(10):e0241316. doi: 10.1371/journal.pone.0241316 (PMC7595327; doi:10.1371/journal.pone.0241316)

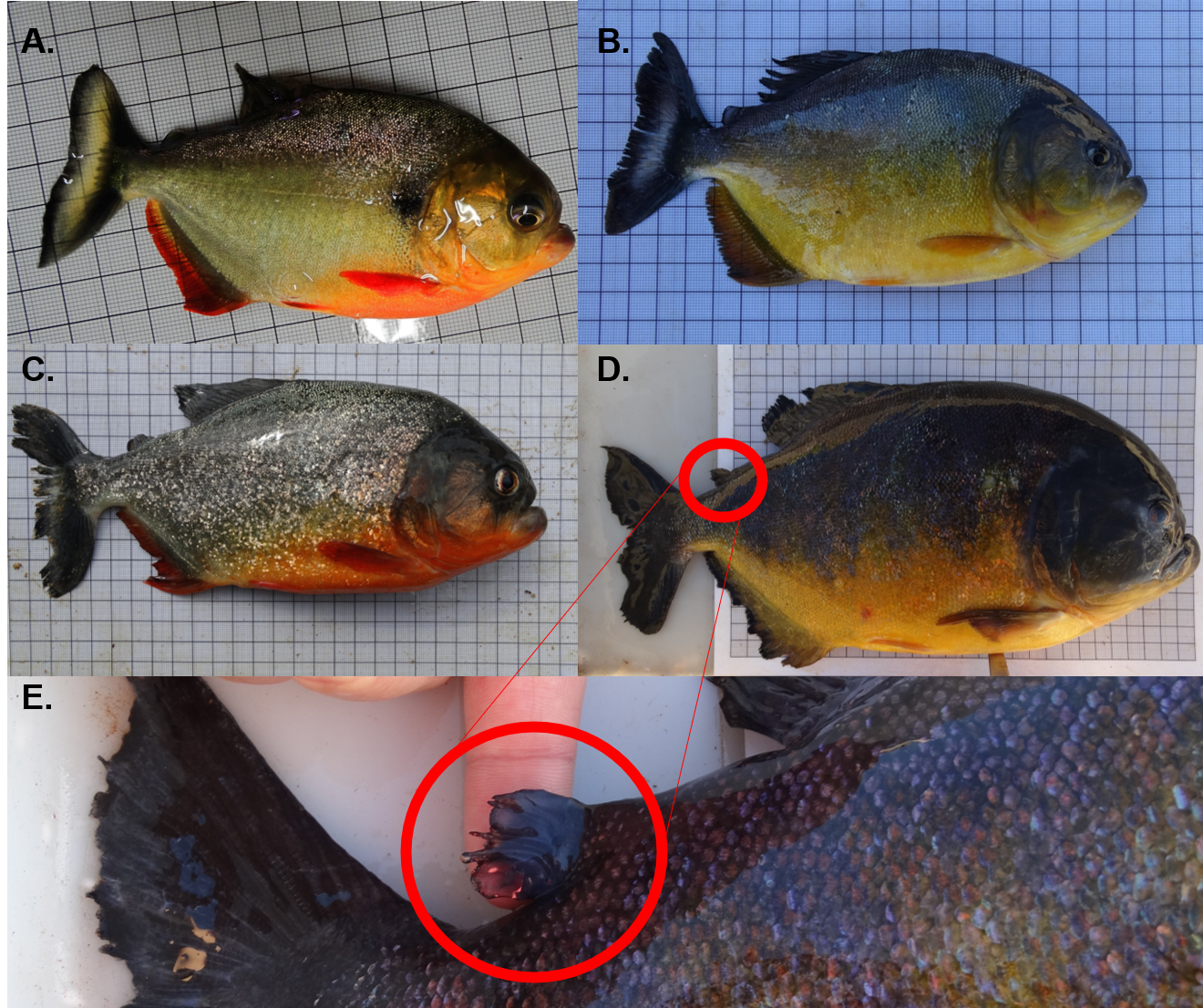

Supplement: S1 Fig — Pictures of a studied specimen of Pygocentrus cariba (A), P. piraya (B), red-bellied P. nattereri (C) and yellow-bellied P. nattereri (D) and a magnification of its adipose fin showing adipose fin rays (E). Millimetre paper (10 x 10 mm) as scale. (TIF) [file pone.0241316.s001.tif]

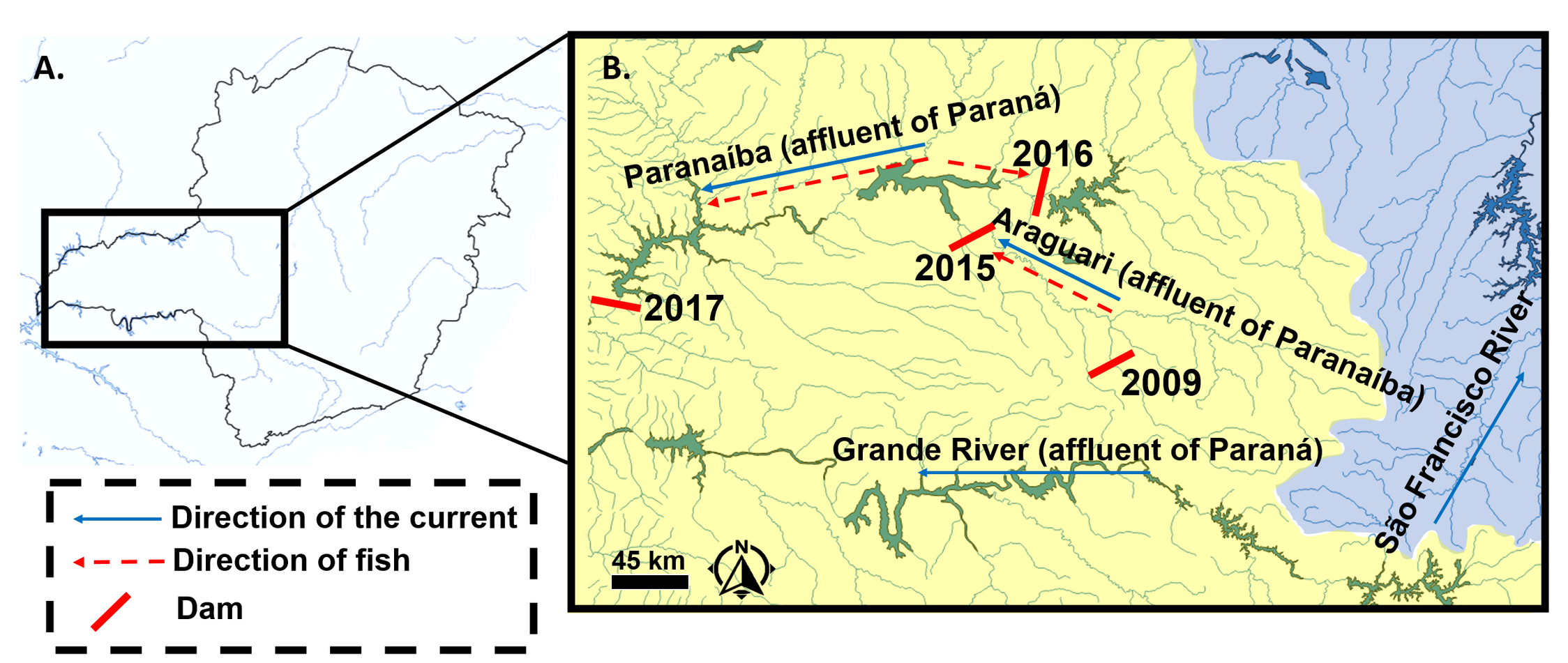

Supplement: S2 Fig — A. Minas Gerais and B. São Francisco River basin (in blue) and Paraná River basin (in yellow) with the sites and dates of first observations of Pygocentrus in the upper Paraná River basin. (TIF) [file pone.0241316.s002.tif]
