# Supplementary material for: Use of bioacoustics in species identification: Piranhas from genus Pygocentrus (Teleostei: Serrasalmidae) as a case study
Source: PLoS One. 2020 Oct 29;15(10):e0241316. doi: 10.1371/journal.pone.0241316 (PMC7595327; doi:10.1371/journal.pone.0241316)
Supplement: S1 Table — SD = standard deviation, SE = standard error, IQR = interquartile range, CV = coefficient of variation, Min = minimal value, Max = maximal value. Acoustic features symbols as defined in Fig 2. Body size (standard length) is given under each species name. (DOCX) [file pone.0241316.s003.docx]

| **Acoustic Feature** | **Statistic** | ***Pygocentrus cariba*** | ***Pygocentrus piraya*** | ***P. nattereri*** | |
| --- | --- | --- | --- | --- | --- |
|  |  |  |  | **Red-bellied** | **Yellow-bellied** |
|  |  | **90 ± 2 mm** | **128 ± 61 mm** | **178 ± 24 mm** | **328 ± 18 mm** |
| **d** (ms) | Mean | 183.40 | 123.07 | 147.22 | 194.58 |
|  | SD | 74.37 | 47.76 | 43.14 | 89.64 |
|  | SE | 14.58 | 4.78 | 1.11 | 8.67 |
|  | IQR | 49.55 | 55.12 | 49.62 | 128.8 |
|  | CV | 0.40 | 0.39 | 0.29 | 0.46 |
|  | Min | 53.70 | 62.70 | 23.50 | 40.00 |
|  | Median | 182.10 | 105.35 | 144.00 | 189.00 |
|  | Max | 388.50 | 265.50 | 417.2 | 465.5 |
| **N** | Mean | 16.23 | 8.78 | 10.60 | 6.20 |
|  | SD | 6.80 | 2.56 | 3.37 | 3.00 |
|  | SE | 1.33 | 0.26 | 0.09 | 0.29 |
|  | IQR | 4.25 | 3 | 4 | 3 |
|  | CV | 0.42 | 0.29 | 0.32 | 0.48 |
|  | Min | 4.00 | 6 | 3 | 1 |
|  | Median | 15.50 | 8 | 10 | 6 |
|  | Max | 34.00 | 18 | 29 | 16 |
| **p** (ms) | Mean | 11.38 | 14.21 | 14.24 | 32.80 |
|  | SD | 0.68 | 4.60 | 2.78 | 8.81 |
|  | SE | 0.13 | 0.46 | 0.072 | 0.85 |
|  | IQR | 0.58 | 2.54 | 3.15 | 10.66 |
|  | CV | 0.59 | 0.32 | 0.20 | 0.27 |
|  | Min | 10.34 | 9.93 | 7.83 | 16.60 |
|  | Median | 11.29 | 12.07 | 13.75 | 31.06 |
|  | Max | 13.43 | 25.95 | 41.50 | 60.75 |
| **d_ez_** (ms) | Mean | 93.87 | 30.69 | 45.10 | 35.20 |
|  | SD | 63.22 | 28.35 | 29.79 | 46.65 |
|  | SE | 12.40 | 2.83 | 0.77 | 4.51 |
|  | IQR | 70.93 | 28.55 | 35.50 | 63.75 |
|  | CV | 673.54 | 923.90 | 660.56 | 1325.40 |
|  | Min | 11.20 | 0.50 | 0.50 | 1.00 |
|  | Median | 87.20 | 23.50 | 39.85 | 1.00 |
|  | Max | 270.00 | 133.00 | 285.00 | 189.50 |
| **N_ez_** | Mean | 7.42 | 3.33 | 4.40 | 1.92 |
|  | SD | 4.57 | 2.28 | 3.37 | 1.27 |
|  | SE | 0.90 | 0.23 | 0.06 | 0.12 |
|  | IQR | 3.75 | 2 | 2 | 2 |
|  | CV | 0.62 | 0.68 | 0.54 | 0.66 |
|  | Min | 2.00 | 1 | 1 | 1 |
|  | Median | 6.50 | 3 | 4 | 1 |
|  | Max | 18.00 | 12 | 23 | 7 |
| **p_ez_** (ms) | Mean | 11.36 | 13.80 | 13.18 | 30.89 |
|  | SD | 0.82 | 4.68 | 2.33 | 11.80 |
|  | SE | 0.16 | 0.47 | 0.06 | 1.14 |
|  | IQR | 0.44 | 2.75 | 2.50 | 12.75 |
|  | CV | 72.55 | 0.34 | 0.18 | 0.38 |
|  | Min | 10.39 | 9.54 | 9.40 | 12.00 |
|  | Median | 11.12 | 11.92 | 12.90 | 30.33 |
|  | Max | 14.70 | 27.75 | 35.57 | 88.50 |
| **F_0_** (Hz) | Mean | 88.89 | 80.32 | 78.86 | 69.75 |
|  | SD | 6.46 | 11.92 | 11.97 | 9.32 |
|  | SE | 1.27 | 1.19 | 0.31 | 0.90 |
|  | IQR | 5.30 | 8 | 15 | 16 |
|  | CV | 0.07 | 0.15 | 0.15 | 0.13 |
|  | Min | 62.70 | 50 | 19 | 52 |
|  | Median | 90.05 | 78 | 78 | 70 |
|  | Max | 96.06 | 105 | 113 | 90 |
